# Optical and Mass Flow Sensors for Aiding Vehicle Navigation in GNSS Denied Environment

**DOI:** 10.3390/s20226567

**Published:** 2020-11-17

**Authors:** Mohamed Moussa, Shady Zahran, Mostafa Mostafa, Adel Moussa, Naser El-Sheimy, Mohamed Elhabiby

**Affiliations:** 1 Department of Geomatics Engineering, University of Calgary, Calgary, AB T2N 1N4, Canada; shady.zahran1@ucalgary.ca (S.Z.); mostafa.mostafa@ucalgary.ca (M.M.); amelsaye@ucalgary.ca (A.M.); 2 Department of Electrical and Computer Engineering, Port-Said University, Port-Said 42523, Egypt; 3 Public Works Department, Ain Shams University, Cairo 11517, Egypt; mmelhabi@ucalgary.ca

**Keywords:** inertial navigation system, optical flow, mass air flow, differential odometry

## Abstract

Nowadays, autonomous vehicles have achieved a lot of research interest regarding the navigation, the surrounding environmental perception, and control. Global Navigation Satellite System/Inertial Navigation System (GNSS/INS) is one of the significant components of any vehicle navigation system. However, GNSS has limitations in some operating scenarios such as urban regions and indoor environments where the GNSS signal suffers from multipath or outage. On the other hand, INS standalone navigation solution degrades over time due to the INS errors. Therefore, a modern vehicle navigation system depends on integration between different sensors to aid INS for mitigating its drift during GNSS signal outage. However, there are some challenges for the aiding sensors related to their high price, high computational costs, and environmental and weather effects. This paper proposes an integrated aiding navigation system for vehicles in an indoor environment (e.g., underground parking). This proposed system is based on optical flow and multiple mass flow sensors integrations to aid the low-cost INS by providing the navigation extended Kalman filter (EKF) with forward velocity and change of heading updates to enhance the vehicle navigation. The optical flow is computed for frames taken using a consumer portable device (CPD) camera mounted in the upward-looking direction to avoid moving objects in front of the camera and to exploit the typical features of the underground parking or tunnels such as ducts and pipes. On the other hand, the multiple mass flow sensors measurements are modeled to provide forward velocity information. Moreover, a mass flow differential odometry is proposed where the vehicle change of heading is estimated from the multiple mass flow sensors measurements. This integrated aiding system can be used for unmanned aerial vehicles (UAV) and land vehicle navigations. However, the experimental results are implemented for land vehicles through the integration of CPD with mass flow sensors to aid the navigation system.

## 1. Introduction

The Global Navigation Satellite System (GNSS) and Inertial Navigation System (INS) integration is the main integrated navigation component in land vehicles. GNSS provides reliable long-term estimates for position and velocity [1]. However, open sky conditions should be fulfilled [2]. INS provides accurate short-term position, velocity, and attitudes estimates for navigation applications [3]. However, this navigation information is degraded over time because of the INS drift due to the sensors (accelerometers and gyroscopes) errors [1], especially when using low-cost INS (MEMS-based) [4]. Therefore, GNSS/INS integration overcomes the drawbacks of each sensor if used in standalone mode and provides a better navigation estimate [5] where GNSS provides position and velocity as updates to the navigation filter, and the INS is responsible for the prediction stage in the integration filter [6].

GNSS/INS integration may suffer in some operating environments such as urban canyons, tunnels, and underground parking [7] where the GNSS signals are degraded or blocked for a long time [8], and the INS only provides the navigation solution, which is deteriorated after a short time because of drifts and biases of accelerometer and gyroscopes [9]. For example, Equation (1) shows the position error due to the INS biases.

(1) $$\delta P=\frac{1}{2}b_{a}t^{2}+\frac{1}{6}b_{\omega }gt^{3}$$

where *δP* is the position error due to the accelerometer bias (*b_a_*), and the gyroscope bias (*b_ω_*), *g* is the gravitational acceleration, *t* is the time of operation in standalone mode. 

Therefore, INS should be aided by other sensors to reduce the drift of the final navigation solution. Odometers [10], magnetometers [11], ultrasonic sensors [12], light detection and ranging (LIDAR) [13], radio detection and ranging (RADAR) [14], and cameras [15] are examples of the aiding sensors that can be used for aiding INS. Moreover, land vehicle motion constraints can also be applied to aid the INS during GNSS signal outages [16]. Non-holonomic constraints (NHC), Zero velocity UPdaTe (ZUPT), and Zero Integrated Heading Rate (ZIHR) [17] are examples of the motion constraints. Finally, maps may be used to aid the land vehicle navigation [18] to reduce the INS large drift. However, some drawbacks are accompanied when using these sensors.

Odometer is the most common sensor that provides velocity information for land vehicles. Unfortunately, some errors affect the precision of the estimated forward velocity using a regular encoder. The wheel, the road surface, and the encoder itself are three sources of odometer errors. The wheel misalignment and the unequal wheel diameter are examples of the errors related to the wheel [19]. The road surface may affect the estimated forward velocity using odometers such as slippery surfaces due to ice or rain. Mountainous roads may cause wheel slipping and skidding [20]. The encoder resolution and sampling rate may affect the precision of the estimated velocity [19]. 

Magnetometer aids the INS to mitigate its heading drift during GNSS signal outages by providing heading updates. However, the estimated heading may be affected by the magnetic interference of the surrounding environment [21], especially in indoor scenarios (e.g., underground parking) for land vehicles. Ultrasonic sensors are typically used for obstacle avoidance [22] in land vehicles. However, [23] used the ultrasonic sensor as an aiding sensor by providing velocity and change of heading updates [24] to the navigation filter. Nevertheless, the sensor installment process and the estimated velocity and the heading change precision are the major drawbacks when using ultrasonic in land vehicle navigation. 

LiDAR is used in environmental perception and navigation in land vehicles [25]. Unfortunately, there are major drawbacks related to their high price, the computational and processing cost, and the surrounding environment effects (i.e., the LiDAR measurements are affected by the dynamic objects), which lead to mismatching issues. Moreover, LiDAR data is affected by rainy weather. RADAR is usually used for collision avoidance [26], lane detection [27] as well as navigation in land vehicles though the high computational costs and high power consumption are major RADAR problems. 

In the case of indoor scenarios, a beacon-based navigation [28,29] system may be used as aiding sensors for land vehicles [30]. However, additional hardware setups for the beacons transmitters and receivers is one of the main drawbacks of using such a system. Vision sensor is used in land vehicles as environmental awareness. Vision can also contribute in the navigation solution either by vision-based [31] or vision-aided navigation system [16]. Visual odometry (VO) is based on the camera motion estimation between successive images [32], where it is implemented by using a single vision sensor or stereo cameras [33]. 

Optical flow is one of the VO approaches, which is based on the bird and insects flight navigation [34]. The optical flow sensors are typically employed for platform velocity estimation. Previous researches integrated VO with INS [35], and/or magnetometers [34], or fused with motion constraints to aid INS [16]. VO can be integrated with maps, for example; [36] proposed a geo-localization land vehicle system based on visual odometry and public domain map sources for GNSS denied environment. A similar land vehicle navigation system for the urban environment was proposed by [37], where stereo VO using weighted non-linear estimation is implemented and fused with digital maps in which probabilistic map matching is used to limit the VO errors. A VO is proposed by [38] using the rear parking camera of land vehicles and integrated with the GNSS to provide an enhanced navigation solution. Reference [39] has proposed a tightly coupled integration between GNSS and visual information from a single sky pointing camera. The visual information is used to detect the open sky condition for GNSS and to estimate the ego-motion of the vehicle in the non-open sky regions. The VO is based on estimating the essential matrix in which the oriented fast and rotated BRIEF (ORB) is used for the feature extraction and matching in addition to the random sample consensus (RANSAC) algorithm is implemented for outlier rejection. 

Another research adopting vision aided inertial navigation system for UAV applications is shown in [40], their approach is based on optical flow and machine learning Gaussian process regression (GPR) to enhance the estimation of the vehicle velocity. They accommodate real-time incremental training session during GNSS availability, then during the unavailability of GNSS the GPR attempt to correct the drift in both INS and VO systems. 

There are some challenges for VO in land vehicle navigation. For example, for the camera that is mounted downwards, there are lack of features because of the narrow field of view in addition to the shadowing problems. For the cameras that are mounted facing the vehicle forward direction suffers from the dynamic objects, which lead to false matching. Therefore, a CPD camera that is faced in the upward direction solves some of the challenges regarding the narrow field of view, the shadowing, and the moving objects issues, especially for the indoor environment. Moreover, there are many features in the ceiling for such an environment (underground parking and tunnels) such as ducts and pipelines, which enhance the features detection and matching process. Finally, the vision sensors are not affected by the weather condition in the indoor environment scenarios. 

Differential wheel odometry concept has been employed in many robotics and vehicle researches [41]. The idea is based on the vehicle model where the change of heading is estimated using the velocity estimated from multiple sensors mounted on the latitude (transverse) axis of the vehicle or robot. Different researches implemented the differential wheel odometry using different sensors such as [42] used multiple RADAR sensors, [23] used dual ultrasonic sensor, [43,44] used the anti-lock braking system (ABS) to provide velocity and heading information to the navigation system. CAN (Controller Area Network) bus may provide some useful information on the vehicle dynamics such as the forward velocity and steering angle data. However, the commercial on-board diagnostics (OBD-II) provides the velocity information and does not typically provide the steering angle data unless additional customized hardware and software designs are developed [45]. Previous researches used consumer portable devices (CPD) in many land vehicle applications such as monitoring the driving condition, evaluating the road surface quality [46], telematics [47], roadside signs recognition [48], lane tracking [49,50], mapping [51], and navigation [52]. CPDs are used to provide a full navigation solution as in [53] utilized the iPhone 4 inertial sensors along with the NHC for land vehicle navigation states estimation. Other researches used CPDs in aiding the land vehicle navigation, as in [54], which mounted CPDs on a land vehicle steering wheel to estimate the steering wheel angle by the CPD accelerometers and compute the heading change which is used as an update in the navigation filter to aid the low-cost INS during GNSS signal outage. 

Therefore, searching for a new configuration for the typical aiding sensors (up-looking camera) and new aiding sensors (Mass Flow meter) to assist the low-cost INS during GNSS signal outage to improve the land vehicle navigation solution. 

Section 2 describes the system overview for the proposed integrated aiding navigation system that is composed of a monocular camera and mass air flow sensors then the integration scheme is illustrated along with different integration navigation schemes (measurements based and federated based fusion techniques). Section 3 discusses the experimental results where the velocity and heading change estimation results are described along with the navigation solution. Finally, the conclusion results are illustrated. 

## 2. System Overview

An integrated system is proposed to aid the INS during GNSS signal outage, especially in indoor scenarios such as underground parking and tunnels. The aiding system is composed of a CPD camera and multiple mass flow meters to provide the navigation filter with both velocity and change of heading information. The next subsections describe in detail the used aiding sensors in the proposed integrated system. 

### 2.1. Monocular Visual Odometry 

A VO is proposed using an up-looking CPD camera where X and Y optical flows are extracted through feature detection by Speeded Up Robust Features (SURF) detector and M-estimator Sample Consensus (MSAC) algorithm for outlier detection. 

The camera is mounted in the up-looking direction and parallel to the ceiling. This setup avoids the dynamic objects in the camera scene and simplifies the velocity estimation by alleviating roll and pitch compensation. This setup uses the ceiling features such as pipes/ducts but suffers from the abrupt illumination changes due to the lights. The optical flow equations are described in [55] as shown in Equation (2).

(2) $$p=\frac{f}{Z}P$$

where *P* is the space point with coordinate (*X,Y,Z*) and *p* is the image point with (*x,y,f*) coordinates, and *f* is the focal length of the camera. *Z* is the range between the camera and the ceiling of the underground parking or tunnel, which may be estimated using an ultrasonic sensor. The image features displacements are multiplied by the observation rate to determine the optical vectors. MSAC is used to choose a single representative optical vector (*u, v*) based on observation consensus. Then the velocity is calculated in Equation (3) where s is the pixel size.

(3) $$V_{VO}=\frac{-s}{f}uZ$$

### 2.2. Mass Flow Sensors 

The mass flow sensor is used to measure the flow rate of the air and gases. It is typically used in laboratory and medical applications. In this paper, multiple mass flow sensor measurements are manipulated to estimate the forward velocity and the change of heading of land vehicles to aid the INS in indoor environments such as tunnels and underground parking where GNSS signal is not available. This section is divided into two subsections where the first one describes the forward velocity estimation using the mass flow sensors while the second subsection illustrates the land vehicle heading estimation using the proposed mass flow differential wheel odometry 

#### 2.2.1. Mass Flow Velocity Estimation 

**Figure 1:**
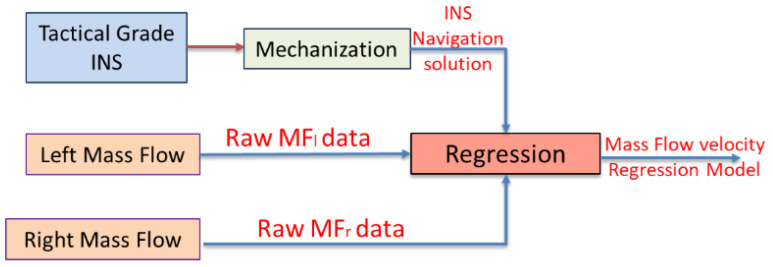
Flowchart of the mass flow velocity regression model estimation.

The relation between the multiple mass flow sensors measurements and the computed velocity from a reference tactical grade INS is estimated through a linear regression model as shown in Figure 1. The mass flow velocity regression model is expected in an indoor environment to avoid the effect of the wind for outdoor scenarios. 

**Figure 2:**
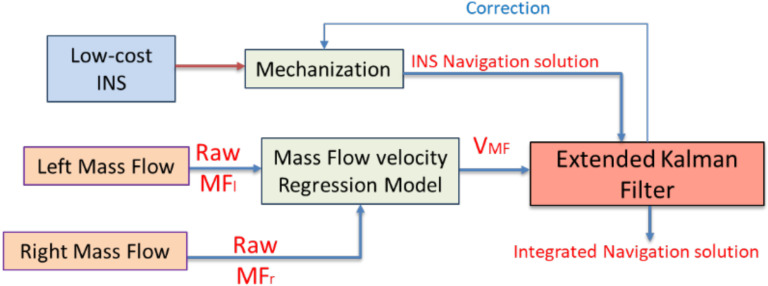
Flowchart of the proposed mass flow velocity aiding low-cost Inertial Navigation System (INS) through extended Kalman filter (EKF).

For indoor scenarios, the multiple mass flow sensors use the predetermined regression model to estimate the land vehicle forward velocity to aid the low-cost INS for mitigating its large drift. Figure 2 exhibits the forward velocity estimation by multiple mass flow sensors and its integration with low-cost INS during GNSS signal outage. 

#### 2.2.2. Mass Flow Heading Change Estimation 

**Figure 3:**
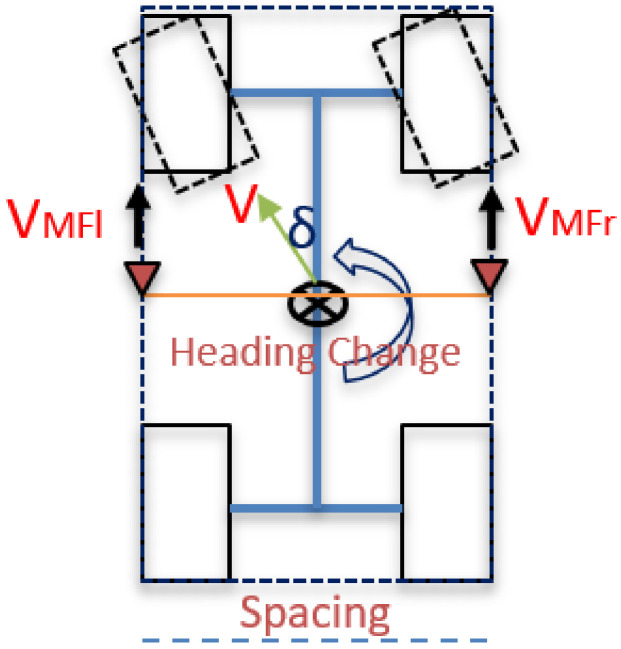
Proposed differential mass flow odometry concept.

The change of heading computation is based on the differential wheel odometry, which depends on the vehicle model concept [56]. Figure 3 and Equations (4)–(6) describe the heading change computation using multiple mass flow sensors.

(4) $$V_{MFl}=V\cos(\delta )-\frac{Spacing}{2}(Heading\;change)$$

(5) $$V_{MFr}=V\cos(\delta )+\frac{Spacing}{2}(Heading\;change)$$

(6) $$Heading\;change=\frac{V_{MFr}-V_{MFl}}{Spacing}$$

where the velocity (*V*) and the rotation angle (*δ*) is at the vehicle’s center of gravity.

**Figure 4:**
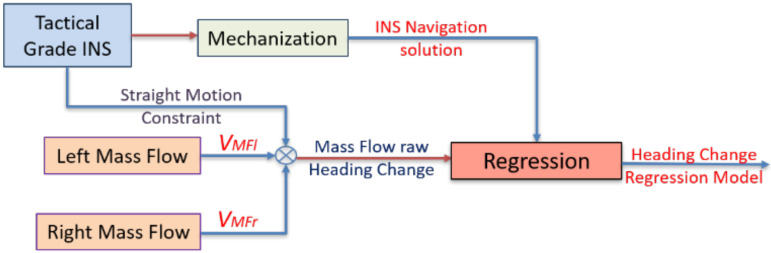
Flowchart of the mass flow heading change regression model estimation.

The land vehicle heading change is estimated by differencing the velocities computed from the left and right mass flow sensors and divided by the spacing between them. The spacing between the multiple mass flow sensors is determined using linear measurements. However, there are two sources of errors when estimating the heading change, which are the two mass flow velocities and the spacing distance. Therefore, the system is calibrated using tactical grade INS through linear regression to compute the bias and the scale factor for the heading computation, as shown in Figure 4. 

**Figure 5:**
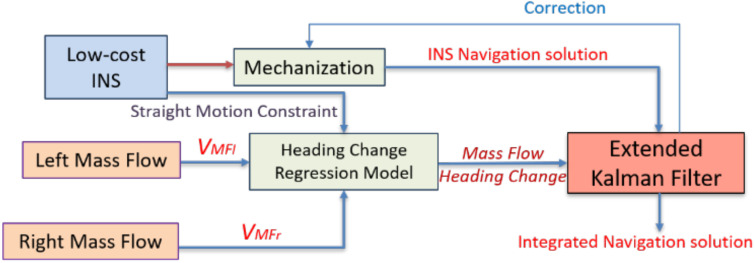
Flowchart of the proposed differential mass flow odometry for aiding low-cost INS through EKF.

The scale factor and the bias are used to compensate for the errors of the heading change estimation using the proposed differential mass flow odometry. Figure 5 exhibits the flowchart of the differential mass flow odometry to aid the low-cost INS during GNSS signal outage. 

**Figure 6:**
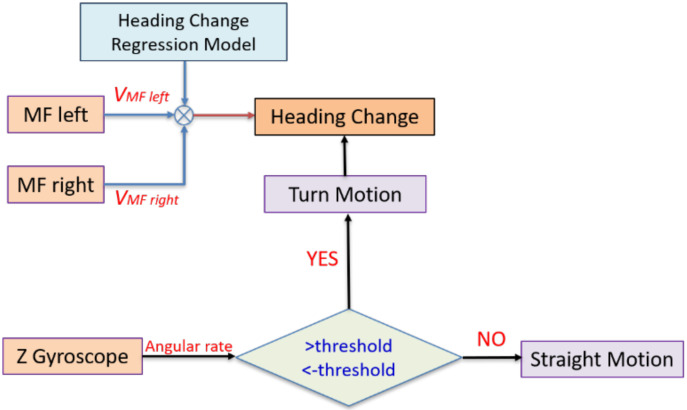
Flowchart of the straight motion constraint using Z gyroscope.

It is worth mentioning that the *Z* gyroscope is integrated with the mass flow sensors in estimating the land vehicle change of heading as it provides a straight motion constraint to the proposed estimating method with the same concept that was described by [23]. Figure 6 shows the flowchart of the straight motion constraint using *Z* gyroscope.

On the one hand, when the *Z* gyroscope angular rate measurements are less than a certain threshold, then the vehicle is in a nearly straight motion, and there is no need to acquire any heading information from the multiple mass flow sensors. On the other hand, when the gyroscope measurements exceed this threshold, then the vehicle is in a turn motion state, and the proposed differential mass flow odometry provides the navigation filter with the change of heading updates. 

### 2.3. Integration Scheme

Two sensors integration techniques are implemented, which are the measurement integration and federated integration methods. On one side, the integration of the measurement is based on fusing the aiding sensors’ observations with the INS in one navigation filter, which is the extended Kalman filter (EKF). 

On the other side, the federated integration scheme depends on integrating the navigation solution where each aiding sensor is fused separately with the INS through banks of KF to provide a navigation solution and then these solutions are fused through weighted average least square adjustment (LSA) to determine one integrated solution. 

During GNSS availability, GNSS/INS loosely coupled integration is used to estimate the navigation solution through EKF. On the other hand, the proposed aiding navigation system provides the navigation filter with the velocity and heading change updates to aid the low-cost INS during GNSS signal outage. 

EKF states include the navigation states’ errors as well as the INS sensor error states (biases and scale factors). The error states vector *δx* consists of 21 states as follows:

$$\delta x_{1\times 21}=\left[\begin{array}{ccccccc} \delta P_{1\times 3} & \delta v_{1\times 3} & \delta \alpha_{1\times 3} & bias_{a_{1\times 3}} & bias_{g_{1\times 3}} & SF_{a_{1\times 3}} & SF_{g_{1\times 3}} \end{array}\right]$$

where *δP*, *δv, and δα* are the position, the velocity, and the attitude error states, respectively. *bias*_a_ and *bias*_g_ are the biases of the accelerometers and the gyroscopes, respectively. Finally, *SF*_a_ and *SF*_g_ are the scale factor of the accelerometers and gyroscopes, respectively. 

KF main phases are the prediction and the update stages [57]. The system model defines the time evolution of the navigation states and counts for the prediction stage while the observation model affords the updates to the navigation filter. 

The system and the prediction stages equations are described in the following equations [57]. 

The system stage:

(7) $$\dot{x}(t)=F(t)x(t)+G(t)w(t)$$

(8) $$x_{k+1}=\phi_{k,k+1}x_{k}+w_{\;k}.$$

(9) $$\phi_{k,k+1}=(I+F\Delta t)$$

(10) $$Q_{k}=E(w_{k}w_{k}^{T}).$$

The prediction stage:

(11) $$x_{k}^{-}=\phi_{k,k-1}{\overset{⌢}{x}}_{k-1}^{+}$$

(12) $$P_{k}^{-}=\phi_{k,k-1}P_{k-1}^{+}\phi_{k,k-1}^{T}+Q_{k-1}$$

where $\dot{x}$ is the state vector rate of change, *F* is the dynamics matrix, *x* is the error state vector, *G* is the shaping matrix, and *w_k_* is the white noise. *ϕ_k,k+1_* is the transition matrix, *I* is the identity matrix, and Δ*t* is the time interval, *Q_k_* is the process noise matrix which describes the uncertainty of the system model. Finally, *P_k_* is the states covariance matrix. (−) refers to the predicted elements, and finally, (+) refers to updated elements [57].

The observation model is described in Equations (13) and (14)

(13) $$z_{k}=H_{k}x_{k}+\eta_{k}$$

(14) $$R_{k}=E(\eta_{k}\eta_{k}^{T})$$

where *z_k_* is the observational vector, *H_k_* is the design matrix, *η_k_* is the measurement noise. *R_k_* is the covariance matrix of the measurement noise, describes the uncertainty of the observations.

When the forward velocity updates *v^b^=[v_MF_ 0 0]^T^* (for example mass airflow velocity updates) are applied to the navigation filter, the observation model and the design matrix are described in Equations (15) and (16)

(15) $$\delta z_{V_{MF}}={\left(C_{b}^{l}\right)}^{-1}v^{l}-v^{b}$$

(16) $$H_{V_{MF}(3\times 21)}=\begin{array}{cccc} {}[0_{3\times 3} & {\left(C_{b}^{l}\right)}^{-1}{}_{3\times 3} & -{\left(C_{b}^{l}\right)}^{-1}{\left[v^{l}\times \right]}_{3\times 3} & 0_{3\times 12} \end{array}]$$

where *v^b^* is the velocity update vector while is *v_MF_* is the estimated forward velocity from the Mass Flow sensors, $C_{b}^{l}$ is the rotational matrix between the body frame to the local level frame. *v^l^*x is the skew-symmetric matrix of the velocity measured in the local level frame.

On the other side, the heading update observation model and the design matrix are explained in Equations (17) and (18).

(17) $$\delta z_{A_{MF}}=A_{INS}-A_{MF}$$

(18) $$H_{A_{MF}(1\times 21)}=[\begin{array}{cccccc} 0_{1\times 3} & 0_{1\times 3} & [0 & 0 & 1] & 0_{1\times 12} \end{array}]$$

where *A_INS_* and *A_MF_* are the headings from the INS and the Mass Flow sensors differential odometry respectively. 

The update stage is illustrated in Equations (19)–(21) [57].

(19) $$K_{k}=P_{k}^{-}H_{k}^{T}{\left[H_{k}P_{k}^{-}H_{k}^{T}+R_{k}\right]}^{-1}$$

(20) $${\overset{⌢}{x}}_{k}^{+}={\overset{⌢}{x}}_{k}^{-}+K_{k}[z_{k}-H_{k}{\overset{⌢}{x}}_{k}^{-}]$$

(21) $$P_{k}^{+}=[I-K_{k}H_{k}]P_{k}^{-}.$$

**Figure 7:**
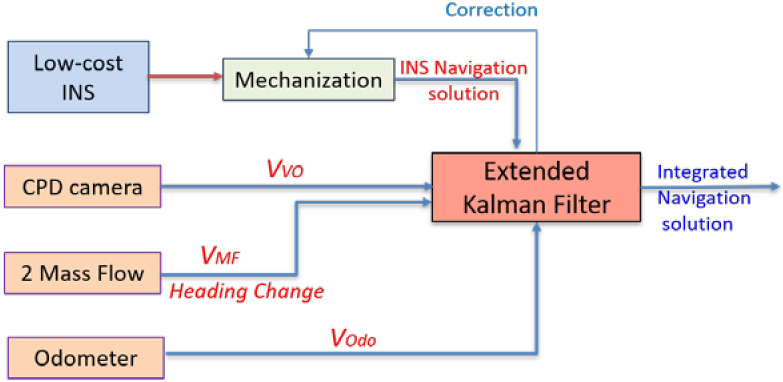
Flowchart of the proposed aiding navigation system measurements integration scheme.

The proposed aiding navigation system consists of an up-looking CPD camera, multiple mass flow sensors, and in-vehicle sensors (odometer). The CPD camera provides the filter with the forward velocity through the optical flow concept. In contrast, the multiple mass flow sensors provide both the velocity and the heading change of the land vehicle. Finally, the vehicle sensors provide velocity update through odometer. These updates are used to aid the low-cost INS to reduce its drift in GNSS denied environment and more specifically, the indoor scenarios such as the tunnels and underground parking. Figure 7 exhibits the flowchart of the proposed aiding system based on the measurement integration scheme. 

**Figure 8:**
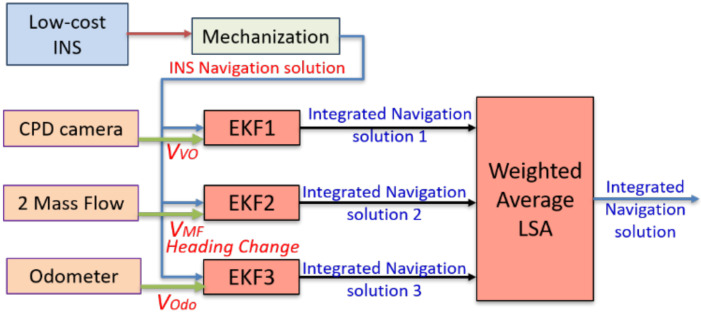
Flowchart of the proposed aiding navigation system federated integration scheme.

Figure 8 shows the proposed aiding navigation system based on the federated integration scheme. Each aiding sensor is integrated separately with INS through EKF to provide a navigation solution then, these navigation solutions, along with their precision (*P* matrix) are fused through weighted average LSA.

The mass flow sensors measurements are modeled using the tactical grade INS from the first beginning. For example, the mass flow regression models are created once. For typical indoor scenario, the CPD camera velocity is regressed using the mass flow estimated velocities for some time before providing the navigation filter with the optical flow speed i.e., estimating the optical flow velocity bias and scale factor for some time from the mass flow sensor, then providing the filter with the regressed optical flow speed. It is essential to mention that the mass flow regression models could be enhanced using the final integrated navigation solution by providing the integrated forward velocity and heading change backward from the EKF.

## 3. Experimental Results

A real data set was collected at the University of Calgary region, where a part of the trajectory was in the underground parking. Pixhawk 2 board is used, which consists of an Invensense MPU-9250 IMU with 50 Hz data rate and a U-blox GNSS with 5 Hz data rate.

**Figure 9:**
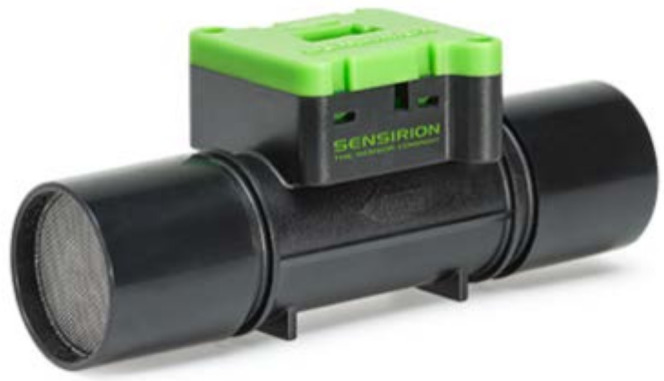
Mass flow sensor (SFM3000).

Two mass flow sensor of the model (SFM3000) and an iPhone 6 camera with up-looking setup were used in the test. The iPhone 6 camera is of model Sony Exmor RS with 1.471 μm pixel size, 29.89 mm focal length, with video resolution 1920 × 1080 pixels and 30 frames per second. The mass flow sensor measure flow rate from range −200 slm to +200 slm (slm is the standard liter per minute, which is a unit of volume flow rate of air corrected to temperature and pressure standard conditions) with operating temperature from −20 °C to +80 °C. Figure 9 exhibits the mass flow sensor used in the experiment

The mass flow sensors were mounted on the roof of the car, and the spacing between the mass flow sensors was 0.99 m. A reference navigation system was used in the experiment to form the linear regression of the mass flow sensors and evaluate the performance of the proposed aiding navigation system. The reference system is based on SPAN technology, which consists of NovAtel OEMV GNSS receiver and tactical grade IMU iMAR FSAS. The tactical grade IMU gyroscopes and accelerometers performance are as follows: The angular random walk is 0.1°/√hr, the rate bias is less than 0.75°/hr. In contrast, the accelerometer bias is 1.0 mg, and the scale factor is 300 PPM for both. The gyroscope input range is ± 500°/s, while the accelerometer range is ±5 g. The position performance is around 4 m RMS and 0.15 m/s for velocity RMS for 60 s GNSS signal outage. Uni-link Mini ELM327 OBD-II Bluetooth Scanner Tool is used to access the regular odometer data. It is worth mentioning here that the SPAN data (SPAN GNSS/INS integrated system) was post-processed through a loosely coupled scheme with EKF employing NHC and ZUPT as updates. Moreover, a forward and backward smoothing process is implemented to estimate the reference solution to help evaluating the proposed navigation system.

### 3.1. Velocity Estimation Results 

The proposed aiding navigation system is based on estimating the forward velocity from different aiding sensors (CPD camera and mass flow sensors). The forward velocity estimated by the CPD camera is based on the optical flow, as discussed before, where the vertical distance (*Z*) between the CPD camera and the underground parking ceiling is pre-surveyed using linear tape measurements. This range may be measured using any ranging sensor such as an ultrasonic sensor.

**Figure 10:**
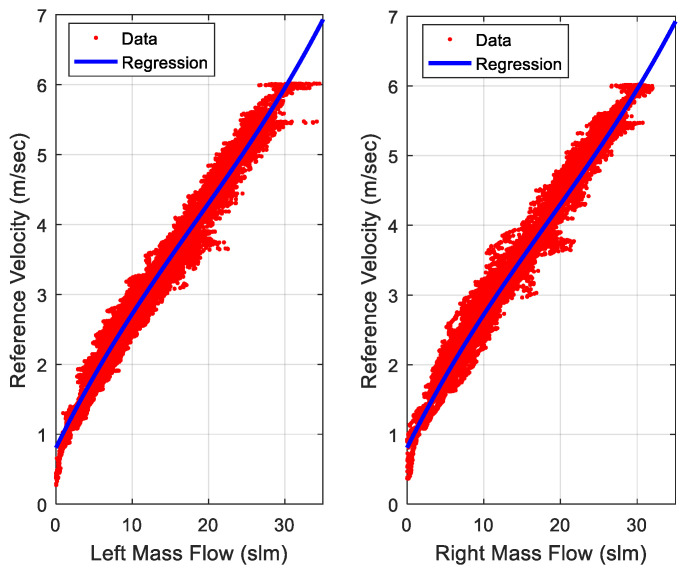
Linear regression between the mass flow sensor measurements and reference velocity estimated by the tactical grade INS.

The mass flow sensors velocity is based on a linear regression model for estimating the relation between the mass flow sensors measurements, and the reference velocity estimated from the tactical grade INS is shown in Figure 10. 

**Figure 11:**
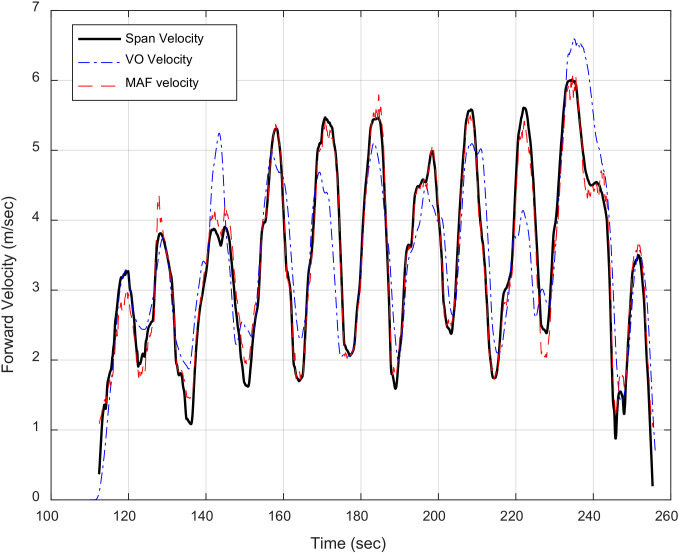
Visual odometry (VO) velocity and mass flow sensors velocity versus SPAN reference velocity.

Figure 11 shows the velocity estimated from different aiding sensors and the reference velocity determined from the tactical grade INS in the underground parking.

The difference between the forward velocity estimated by the VO and mass flow sensors and the SPAN reference velocity is calculated to evaluate the proposed methods. The root mean square error (RMSE) of the forward velocity estimation is 0.62 m/s and 0.27 m/s for the VO and the mass flow sensors methods, respectively.

### 3.2. Heading Change Estimation Results 

The heading change is estimated using multiple mass flow sensors. The proposed method is based on the differential odometry concept, as described before in Equation (8).

**Figure 12:**
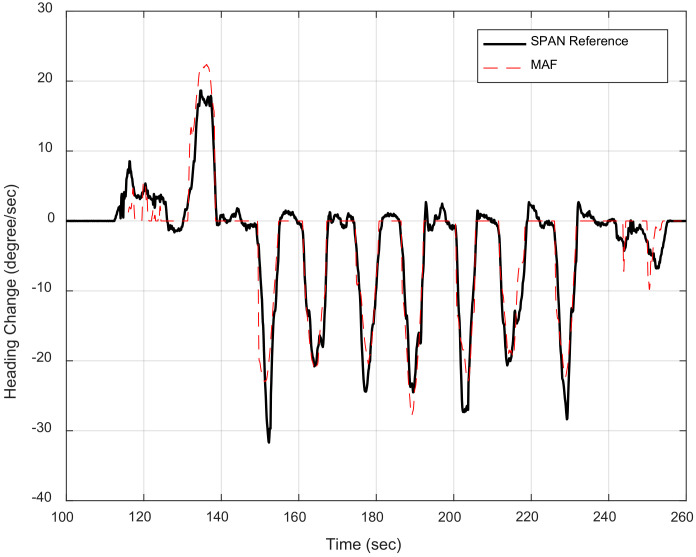
Mass flow change of heading versus SPAN reference heading change.

Figure 12 exhibits the heading change estimated by the proposed method and the reference heading change computed from the SPAN tactical grade INS. 

The difference between the heading change estimated by the mass flow sensors and the SPAN heading change is calculated to assess the proposed differential mass flow odometry method. The RMSE of the heading change estimation is 2.9°/s.

### 3.3. Navigation States Estimation Results 

Loosely coupled GNSS/INS integration is implemented to estimate the navigation solution for the trajectory. The GNSS signal outage occurred for 190 s. when the vehicle entered the underground parking. The position RMSE of the INS stand-alone solution is around 1.98 km. It is worth mentioning that the low-cost INS standalone solution is implemented without any accelerometers and gyroscopes calibration and without the aid of the motion constraints such as Non-Holonomic Constraints (NHC). 

**Figure 13:**
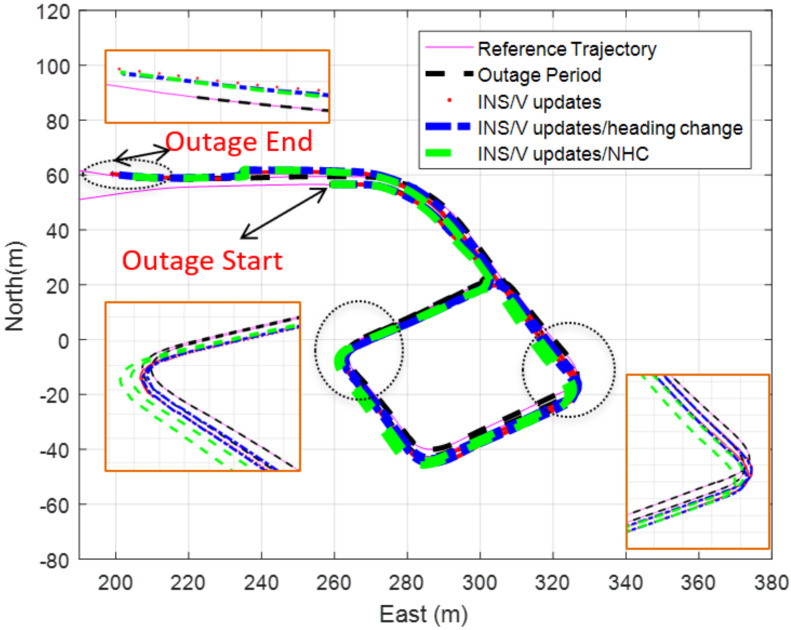
The trajectory of the underground parking using different updates for 190 s. Global Navigation Satellite System (GNSS) signal outage (measurements fusion method).

The navigation states are estimated using different updates to investigate their effect on the final navigation solution, as shown in Figure 13. 

The velocity updates are computed from the VO, mass flow meters, and an odometer, which is based on the measurement’s fusion technique. The difference between the trajectory of the reference and the estimated navigation solution using different updates are computed to calculate the position RMSE to assess the impact of each update on the final result as shown in Table 1 that describes the position RMSE of different navigation solution using various updates by a measurements fusion technique. 

Table 1 shows that aiding the INS with the proposed velocity and heading change updates provide more reliable navigation solution that aid the INS with the regular odometer only during GNSS signal outage as the level of enhancement when using the proposed aiding system reached to around 35% when compared with aiding the INS with the typical odometer. 

The Federated fusion technique is implemented to estimate different navigation solutions using various updates to show the difference between this kind of navigation solution integration and the measurement fusion method. Table 2 shows the position RMSE of different solutions using the federated fusion method. 

**Figure 14:**
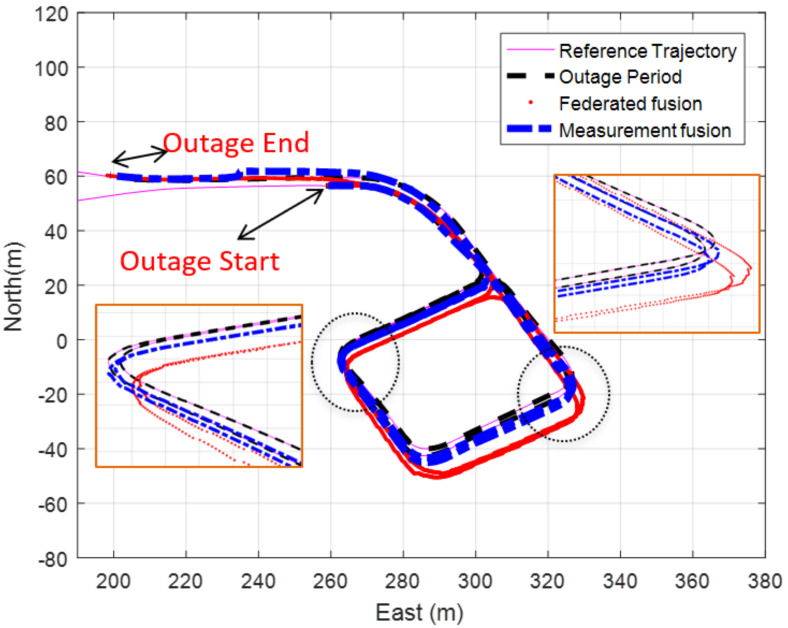
The trajectory of the underground parking INS/velocity/heading change updates for 190 s GNSS signal outage (measurements and federated fusion method).

Table 1 and Table 2 show that the measurement fusion technique provides a better navigation solution than the federated one. The performance difference between the measurement integration and federated integration schemes are mainly due to the mechanization correction step adopted in the measurement integration scheme. Any miss estimation of the position at early epoch(s) will be propagated to the next epochs, which induces this bias effect. Figure 14 exhibits the trajectory of the INS/velocity/heading change updates using both the measurements and the federated fusion methods.

## 4. Conclusions

A multi-modal aiding navigation system based on optical and mass air flow is proposed to aid the low-cost INS in autonomous vehicle navigation for the indoor environment. The proposed system consists of an up-looking camera (CPD) and multiple mass flow sensors where the camera provides forward velocity information through VO while mass flow sensors provide the navigation filter with both velocity and change of heading updates. 

The up-looking camera overcomes the lack of features issues for indoor environment in addition to avoiding the dynamic objects, which makes the features detection and matching more reliable. Redundant velocity information for the land vehicle navigation from different sensors is implemented for fault detection and identification to overcome some of the odometers’ drawbacks.

The proposed aiding system provides the heading change information, which is not affected by magnetic interference as the case of the magnetometers. Moreover, the estimated velocity from the mass flow sensors is based on the quantity of air that passes through the sensors due to the land vehicle motion and therefore overcomes the slipping and skidding problems facing the regular wheel odometer and therefore the mass flow sensors better reflect the car motion.

The proposed aiding navigation system may be applied for both land vehicles and unmanned aerial vehicles, especially for the up-looking camera for indoor rescue. The mass flow sensors provide both velocity and heading change with a high data rate up to 120 Hz with good accuracy for the velocity and performance for the heading change, which can be helpful for some applications that face the challenge of harsh dynamics. 

The cost of the mass air flow sensors is relatively higher than the other sensors (around 200 dollars) which could be reduced if massively produced for land vehicle navigation. Moreover, extreme light variations of the ceiling may deteriorate the optical flow accuracy.

A land vehicle experiment was conducted, and the results showed that the proposed aiding system enhances the navigation solution dramatically compared with the INS stand-alone solution. Different fusion schemes were implemented, which are the measurement integration scheme and the federated scheme, and the results showed that the measurements based provide a better solution than the federated one.

## Figures and Tables

**Table 1:** Position root mean square error (RMSE) for different navigation solution methods using measurements fusion technique.

Navigation Solution	RMSE (m)
INS stand-alone	1980
INS/non-holonomic constraints (NHC)	14.30
INS/odometer velocity update	4.85
INS/velocity update	3.96
INS/velocity/NHC	4.00
INS/velocity/heading change	3.60

**Table 2:** Position RMSE for different navigation solution methods using federated fusion technique.

Navigation Solution	RMSE (m)
INS stand-alone	1980
INS/NHC	14.30
INS/velocity update	7.94
INS/velocity/NHC	6.07
INS/velocity/heading change	6.74
